# Daily arousal variation has little effect on sustained attention performance

**DOI:** 10.1007/s12144-023-04473-9

**Published:** 2023-03-17

**Authors:** Isobel G. Bond, Keitaro Machida, Katherine A. Johnson

**Affiliations:** Melbourne School of Psychological Sciences, University of Melbourne, Parkville, Victoria 3010 Australia

**Keywords:** Sustained attention, Arousal, Time-of-day, Inverted-u, Small-n

## Abstract

Sustaining attention is an important cognitive process for everyday functioning and arousal is thought to underpin its performance. Primate studies depict an inverted-u relation between sustained attention and arousal, in which sustained attention performance is most affected at the extreme levels of arousal and peak performance aligns with moderate arousal. Human research findings are, however, inconsistent. This study aimed to investigate the effects of arousal on sustained attention performance in humans using two approaches—a small-*N* study with an inbuilt replication to test within-participant variation, and a larger sample assessing between-participant variation. The Sustained Attention to Response Task (SART) was used to measure sustained attention performance and the Karolinska Sleepiness Scale (KSS) was used to measure arousal. In the small-*N* study five participants completed the SART and KSS once an hour between 7 a.m. and 7 p.m., repeated two weeks later. Significant, curvilinear variation in KSS across time-of-day was found. A linear association between SART response time variability (sigma) and KSS was noted, however no other consistent associations between the SART and KSS were found. In the large-*N* study, 161 participants completed the SART and KSS once, at a time of day of their choosing. There were no significant relations between SART measures and the KSS, indicating that subjective sleepiness was not related to sustained attention performance. Overall, the hypothesized inverted-u relation between arousal and sustained attention performance was not found. The results suggested that diurnal arousal variation does not modify sustained attention performance in adults.

Sustained attention is the ability to allocate attentional focus towards a repetitive stimulus (Robertson et al. 1997) and is essential for daily functioning (van der Heijden et al., 2010). The ability to maintain attention is dependent on sufficient levels of cortical arousal (Esterman & Rothlein, 2019; Lenartowicz et al., 2013). Arousal is the subjective experience of being awake and ready to think and act (Perrin et al., 2019), and is often measured in humans using self-report scales of sleepiness, such as the Karolinska Sleepiness Scale (Åkerstedt & Gillberg, 1990). Variation in arousal shows an inverted-u pattern across the course of the day in which the aroused state is highest in the middle of the day, and this is consistent in the literature (Åkerstedt et al., 2013; Anderson & Revelle, 1994; Manly et al., 2002). Arousal is underpinned by noradrenaline, a product of the locus coeruleus (Aston-Jones et al., 2007; Unsworth & Robison, 2017; Valdez, 2019). In monkeys and rats, an inverted-u shape describes the relation between sustained attention task performance and levels of locus coeruleus activation (Aston-Jones & Cohen, 2005; Berridge & Waterhouse, 2003). At the two extremes of locus coeruleus activation—states of hypo- and hyper-arousal—sustained attention performance was at its worst (Aston-Jones & Cohen, 2005; Berridge & Waterhouse, 2003). In humans, the association between sustained attention performance and arousal, measured as sleepiness, is less clear in the literature. Research on the pattern of performance on sustained attention tasks at different times of the day, and concomitant varying sleepiness levels is equivocal. Further research is needed to explore how sustained attention varies across sleepiness levels and time-of-day. This is important for work planning, safety, educational policy, and for understanding participant performance within experimental research paradigms.

Tasks often used to measure sustained attention include variations of the Sustained Attention to Response Task (SART) (Robertson et al., 1997) and the Continuous Performance Task (e.g. the gradCPT) (Fortenbaugh et al., 2015). In these tasks the participant is asked to respond to the specified “go” targets and refrain from responding to the “no-go” targets. Commission errors represent a response made to no-go targets, omission errors reflect absence of response to go stimuli, and *d’* and criterion are indices of target sensitivity and response bias respectively. These measures, together with speed and variability of response are all common measures of sustained attention performance (Machida et al., 2022).

Whether sustained attention performance varies depending on participant sleepiness and time of day is a matter of debate. The most comprehensive study on this question, to date, is the Manly et al. (2002) paper. They asked a small sample of ten university students to complete a nine-minute version of the SART, every six hours (1 a.m., 7 a.m., 1 p.m., 7 p.m.), for four days, while maintaining their daily routines. As expected, an inverted U-shape curve was found for sleepiness, with participants feeling sleepy at 1am and most sleepy at 7am, and most awake at 1 pm and 7 pm. A linear trend in commission error performance was found across the day, with most errors made at 1am, then at 7am, and better performance at 1 pm and 7 pm. The number of commission errors correlated with sleepiness. In contrast, the speed of response did not vary across the day and did not correlate with sleepiness. The authors suggested that sleep/circadian disruption disproportionately affected cognitive control (e.g., commission errors) compared with more automated responses (e.g., reaction times).

Other studies of association between sustained attention performance and sleepiness report contradictory results. Stawarczyk and D'Argembeau (2016) contended that sleepiness impaired sustained task performance in a linear fashion. Decreased target accuracy and greater variability in responding correlated with increasing sleepiness in a long duration, mind-wandering probe version of the SART. The time of day of testing was not noted, however. In direct contrast, van Schie et al., 2014 found no association between sustained attention performance and sleepiness, where adults completed the SART at two 1.5-h intervals in either the morning or the afternoon. The greater difficulty of the task (Stawarczyk & D'Argembeau, 2016) compared with optimal (during the day) time of testing (van Schie et al., 2014) may explain these differences in results.

Time-of-day appears to influence sustained attention performance. van Schie et al. (2014) reported no significant differences in SART error performance between the morning and afternoon groups, but responses were slower in the morning than in the afternoon. Riley et al. (2017) found time-of-day effects for commission errors, *d’*, coefficient of variation in reaction time, and reaction time, but not for omission errors or criterion. Participants performed optimally between 9 and 11am, with a slow reduction in performance across the day, and a more rapid deterioration in performance between 11 pm and 5am. Reaction time was slowest at midday and fastest at midnight, but the differences across the day were small. This was a large (*n* = 6,363), online, independent measures study measuring sustained attention using the gradCPT task, with measures taken at any time of the day; no measure of sleepiness was taken however (Riley et al., 2017). In contrast with the Manly et al. (2002) study, there was a subtle but significant diurnal variation in reaction time, with slowest responses made between 12 p.m. and 4 p.m. Extrapolating knowledge of an inverted-u progression of arousal levels across the waking day (Anderson & Revelle, 1994; Facer-Childs et al., 2018), Riley et al. (2017) indirectly suggests the possibility of a curvilinear relation between sleepiness and sustained attention. A summary of this past research suggests that more difficult tasks and times of testing outside of morning and afternoon result in worse sustained attention performance, with commission errors and response time variability being more affected by sleepiness than mean response time and omission errors.

Further research into daily arousal variation and SART performance is warranted. Knowledge of how sustained attention performance and sleepiness co-vary, especially within typical ranges of arousal and working hours, has real-world implications for anyone completing tasks that require focus over time. The research reported here specifically focused on arousal variations that individuals experience in daily life rather than extreme arousal levels created by medication or sleep deprivation.

To illustrate better the relation between sleepiness and sustained attention performance, two research designs were used in two separate studies. One of the major reasons psychological studies are often not replicable, or show inconsistent results, is that there are large individual differences (Smith & Little, 2018). These individual differences can be controlled by examining the effect within individuals rather than between individuals; therefore, the first study used a repeated measures within-subject design with a small number of participants to gain a richer sense of the relations between arousal variation, measured using sleepiness, and task performance while reducing between-subject variation. Conversely, relying on within-subject research design might reduce generalisability of the result to the general population. Therefore, the same research question was studied using an independent measures design with a larger group of participants to observe the relationship between sleepiness and sustained attention performance. In Study 1 a small sample of participants performed a sustained attention task thirteen times across a day, over two days, with sleepiness measured at each testing time. In Study 2, a larger sample of participants performed the same task once, with time of day and feelings of sleepiness recorded. The hypothesis in both studies was that sustained attention performance would show an inverted-u relation with levels of sleepiness; time of day was used to provoke variations in sleepiness.

## Study 1: small-***N***

Study 1 was designed to examine within participant effects of sleepiness on SART performance using a small number of participants (Small-N). A small-*N* design facilitates detailed analysis by considering the individual, rather than the experiment, as the unit of replication (Smith & Little, 2018). Testing for the effect of interest within a participant, using multiple sessions, allows contextual and individual variation to be controlled. As outlined by Valdez (2019), time-of-day recordings are typically used to measure diurnal variation in constructs. Their usefulness is limited however by the large samples required to obtain significant differences, inviting high levels of variability into the results. A small*-N* design reduces the individual variability risk through repeated use of a small sample to collect many trials across a range of testing sessions. Arousal’s effect on sustained attention is reflected in an individual’s task performance. For this reason, initially creating a detailed model of this variance within an individual is of greater use than contrasting this variable at different levels across a wide range of participants. This study attempted to measure multiple performances of a multi-trial task from a single participant, assessing the relations between arousal and sustained performance measures whilst controlling individual difference variations. By testing multiple time points within a day, varying arousal levels could be evaluated without requiring an intervention to experimentally manipulate participant arousal. Additionally, several participants were tested to examine if the same relationship could be observed and replicated between participants.

The aim of this study was to use a small sample of participants to measure how SART performance varied with arousal levels across two days of repeated testing. The first hypothesis was that arousal and time-of-day would have a curvilinear relation, with peak arousal at midday and lowest arousal in the early morning and late evening. The second hypothesis was that arousal and SART performance would have an inverted-U relation, where the greatest number of errors and response time variability would occur at low and high levels of arousal.

## Method

### Participants

Five participants (three women, two men) were recruited from within the research team (IB, KM, KJ) and two family members of IB; convenience sampling was used because of the intensive testing. Their ages ranged from 21 to 53 (*M* = 34.1 years, *SD* = 13.2) and all participants were right-handed. Each participant gave informed consent and was debriefed at the conclusion of the study. None of the participants reported having existing or previous mental health conditions, neurological conditions, or serious head injuries that would necessitate exclusion from the sample. This study was conducted with the approval of the University of Melbourne Human Research Ethics Committee.

### Measures

#### Demographic questionnaire

Participants responded to demographic questions using a Qualtrics based questionnaire. They were queried about their age, gender, handedness, history of attentional disorders, anxiety, depression, head injury, epilepsy, and information about current medication usage.

#### Attention-Related Cognitive Errors Scale (ARCES)

ARCES is a 12-item questionnaire with responses ranging from 1 (*never)* to 5 (*very often*) used to assess everyday performance errors attributed to failures of sustained attention (Cheyne et al., 2006; Smilek et al., 2010). The ARCES was included to provide a further description of participants but was not included in the analyses.

#### Composite Morningness Questionnaire (CMQ)

CMQ is a 13-question assessment of diurnal preference using scales of one to five for three items and one to four for 10 items, with higher scores indicating greater morning chronotype characteristics (Smith et al., 1989). Validated on a large sample of undergraduate students, evening chronotype is indicated by an overall score of 22 or below and morning chronotype by 44 or above. All other scores are classed as intermediate.

#### Karolinska Sleepiness Scale (KSS)

The KSS is a self-report, nine-point Likert scale (1 = *extremely alert* to 9 = *extremely sleepy, fighting sleep*) to quantify a participant’s sleepiness (Åkerstedt & Gillberg, 1990).

#### Cognitive Interference Questionnaire (CIQ)

The CIQ uses 21 questions to quantify task-related and task-irrelevant cognitive intrusions and mind-wandering that participants might have experienced during a task they just completed (Sarason et al., 1986), using a five-point scale (1 = *never* to 5 = *very often*). The CIQ was included to provide a further description of participants but was not included in the analyses.

#### Sustained Attention to Response Task (SART)

The SART (Robertson et al., 1997) is an experimental computer task consisting of 18 practice trials and 225 trials of the digits 1–9 randomly presented for 250 ms each followed by an ‘x’ backwards mask for 900 ms. Participants were asked to press the space bar with the index finger of their dominant hand when each digit was presented, withholding their response when the digit 3 was presented. Each digit was presented 25 times in white against a black background, in 48-, 72-, 94-, 100-, and 120-point size Symbol font. Inquisit player software was downloaded onto each participants’ own computer to run the SART. In this way computer variation and experimental setup were unique to each participant. The task took approximately five minutes.

To guard against practice effects, the small-n study used a fully random version of the SART. This contrasts with the pseudo-randomised SART used in Study 2, in which a set of digits was arranged so that the no-go digit ‘3’ was never presented twice in a row.

Performance on the SART was indexed by response time (RT) and counts of commission and omission errors. RT measures were additionally fitted to ex-Gaussian distributions to model RT variance as mu, sigma, and tau (Luce, 1986). Mu and sigma are equivalent to the mean and standard deviation of response time, estimated from the Gaussian distribution of RT data, and tau indexes extremely slow RTs and is estimated from the exponential distribution.

### Procedure

All recruitment and experimental procedures occurred online within the participant’s home using a personal laptop device in a distraction-free space, during COVID19-related lockdowns. Days prior to the initial testing session, an introductory online survey, using Qualtrics, was completed that included a plain language statement, consent form, demographic questionnaire, ARCES, and CMQ. Participants were then asked to download Inquisit player onto their computer. They then completed a practice SART in preparation for the testing sessions. The experimental sessions occurred over two days, spaced two weeks apart. Across the day, participants completed the SART once per hour between the hours of 7 a.m. and 7 p.m. Prior to commencing each SART, participants entered their unique participant code and indicated their current arousal level on the KSS. Following the SART, participants completed a second KSS rating and the CIQ. Participants spent approximately seven minutes every hour completing these tasks. On the testing days, participants were otherwise asked to pass the day as usual. Following the initial 13 sessions, participants replicated this exact procedure 14 days later. In this study, one testing session was considered as one sample; this contrasts with typical psychology studies where one participant is considered as one sample. Across all 5 participants, 130 testing sessions were completed. Once all testing sessions were complete, participants were debriefed.

### Data preparation

Questionnaire responses were scored in accordance with their respective guidelines and subsequently imported into RStudio.

#### KSS

During Day One, participants rated feeling more sleepy after completing the SART, before: *M* = 4.42, *SD* = 1.78, after: *M* = 4.75, *SD* = 1.81, *t*(63) = 3.07, *p* = 0.00. In contrast, during Day Two there was no significant difference in KSS before and after the SART, before: *M* = 4.43, *SD* = 1.84, after: *M* = 4.58, *SD* = 1.83, *t*(64) = 1.56, *p* = 0.12. Therefore, an averaged KSS score was calculated to provide an estimate of arousal for each testing session, for each participant.

#### SART data preparation

To generate SART performance outcomes per participant, preparation and analysis of SART data was completed using MATLAB (Johnson et al., 2020). The MATLAB script used is available at the Open Science Framework at https://osf.io/ntwy7/.

Commission and omission errors were counted per participant per SART. Participants were excluded if they made more than 30 omission errors, with the understanding that the participant was not completing the SART appropriately; no data-sets were excluded. The ex-Gaussian measures of mu, sigma, and tau were calculated per participant per SART (Lacouture & Cousineau, 2008; Luce, 1986).

#### Missing data and exclusions

A recording malfunction occurred in a testing session for one participant, hence there was one session missing on the first day of testing. Therefore 129 testing sessions were completed and analysed, 64 on day one and 65 on day two. The five participants each had average ARCES scores (*M* = 2.33, *SD* = 0.14) and were all within the intermediate chronotype range on the CMQ (*M* = 33.56, *SD* = 4.98) hence these measures were not investigated as moderating factors. The number of omission errors made by the participants was very low, therefore no further analyses were conducted with this measure.

### Statistical analysis

R version 3.5.2 (Team, 2018), RStudio Version 1.3.1073, and the *lme4* package (Bates et al., 2015) were used generate linear mixed-effects models (LMEMs) with random intercepts to analyse the data. As commission errors were count data, a generalised LMEM with Poisson distribution was used to model variance of this outcome measure. The data were modelled for the two testing days separately, as an in-built replication. Participant ID was included as a random effect, with the aim to minimise repeated measure effects.

Spearman’s rho correlation coefficients were calculated for each outcome variable against KSS, for each participant by day. These were then visually inspected to illustrate within and between participant differences across the experiment.

To test hypothesis one, time-of-day, time-of-day as a second-order polynomial (as fixed effects) and Participant ID (random effect) were included in an LMEM to explain variance in KSS scores. Separate models were created for each day of testing. To test hypothesis two, four models for each SART performance outcome were created to explain the variance in commission errors, mu, sigma, and tau. These four models comprised linear and quadratic LMEMs for each day of testing. In the linear LMEM, a fixed effect of KSS and random effect of Participant ID was used to explain variance in each SART measure. In the quadratic LMEM, fixed effects of KSS and KSS as a second-order polynomial term (KSS^2^) and Participant ID (random effect) were added to explain variance in each SART outcome. This quadratic model aimed to model the hypothesised inverted-u relation. To compare the four models generated for each outcome variable significance testing, Akaike’s information criterion (AIC), Bayesian information criterion (BIC) were implemented and interpreted. Predictors were considered significant if the 95% confidence interval (CI) of the *b* coefficient did not cross zero for LMEMs or cross one for generalised LMEMs.

## Results

### Descriptive statistics and correlations

**Fig. 1:**
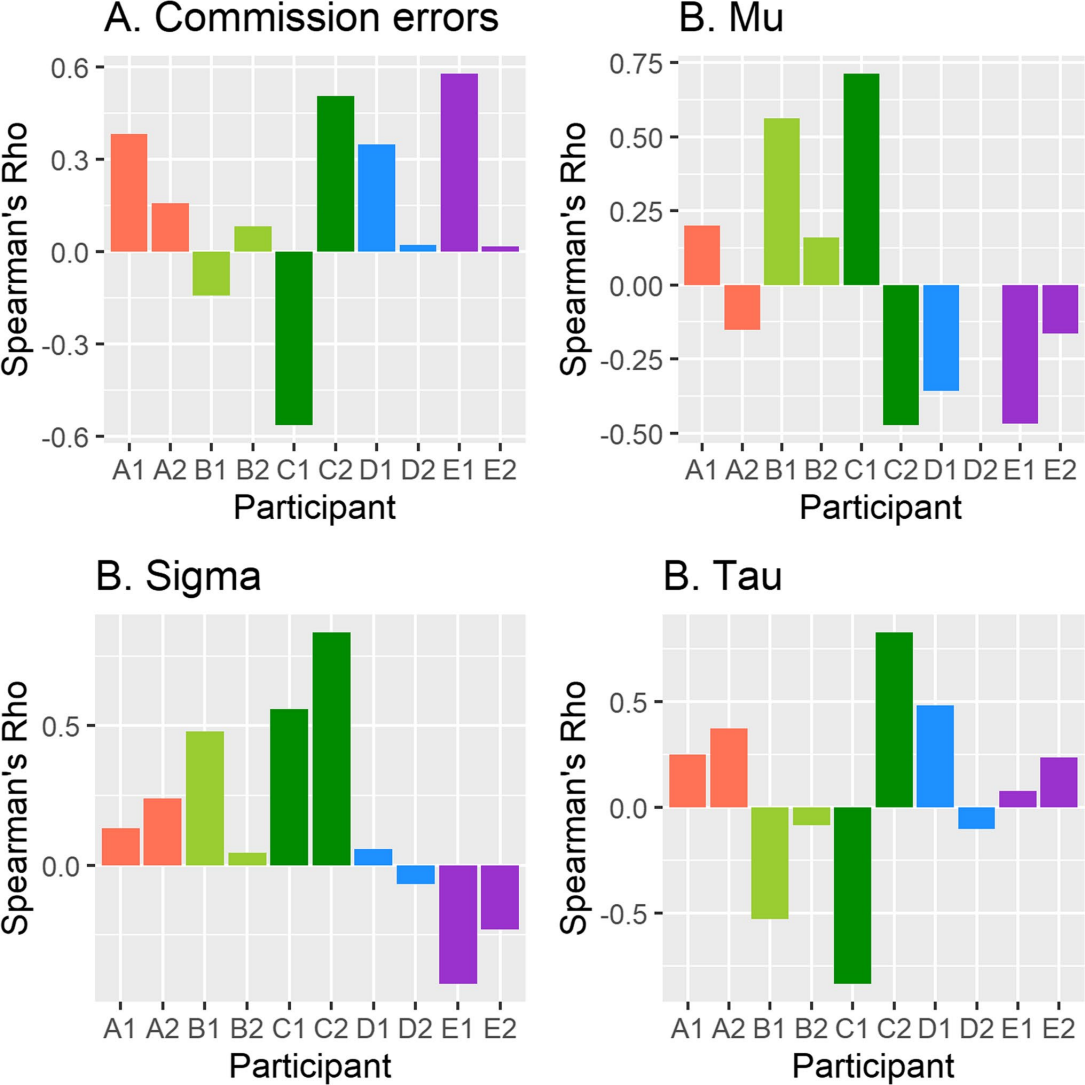
Spearman’s Rho coefficients between each SART measure and KSS for each participant (A to E) on days 1 and 2 (Study 1)

The mean, standard deviation, and score range of participants’ responses to questionnaires and SART performance are presented in Table 1. Correlations were used to observe within-participant differences on the SART measures across the thirteen testing sessions, separately for the two days of testing (see Fig. 1). These correlations were then pooled across participants and testing days, and means and SD were calculated. Small correlations with large variance were noted—commission errors (*M* = 0.14, *SD* = 0.34), mu (*M* = 0.00, *SD* = 0.41), sigma (*M* = 0.16, *SD* = 0.38), and tau (*M* = 0.07, *SD* = 0.49)*.*

**Table 1:** Descriptive statistics of participant responses in Study 1

Measure	Group *Mean (SD)*	Participant A* Mean (SD)*	Participant B* Mean (SD)*	Participant C* Mean (SD)*	Participant D* Mean (SD)*	Participant E* Mean (SD)*
ARCES^a^	2.3 (0.1)	2.5	2.4	2.1	2.3	2.3
KSS	4.5 (1.8)	4.8 (2.0)	3.6 (2.0)	4.4 (1.8)	4.8 (1.6)	5.1 (1.3)
CIQ	1.6 (0.8)	1.6 (0.8)	1.7 (0.9)	1.4 (0.8)	1.5 (0.7)	1.5 (0.9)
Mind Wandering^b^	4.0 (1.2)	3.7 (1.3)	3.5 (1.4)	4.8 (1.0)	4.2 (1.3)	4.0 (0.7)
Commission Errors	7.5 (4.4)	11.9 (3.9)	2.2 (1.5)	8.2 (3.2)	7.9 (2.1)	7.3 (4.3)
MRT (ms)	383 (50)	326 (22)	415 (35)	415 (52)	371 (30)	386 (41)
SDRT (ms)	71 (21)	54 (11)	59 (11)	90 (19)	80 (23)	71 (16)
Mu (ms)	340 (62)	286 (20)	376 (40)	399 (76)	304 (18)	335 (46)
Sigma (ms)	48 (23)	33 (9)	42 (12)	81 (28)	37 (9)	48 (15)
Tau (ms)	43 (27)	40 (10)	40 (13)	17 (30)	67 (23)	51 (24)

^*a*^Attention-Related Cognitive Errors Scale ^b^Item score on CIQ: Mind Wandering

### Hypothesis one: time of day and kss will have a curvilinear relation

**Fig. 2:**
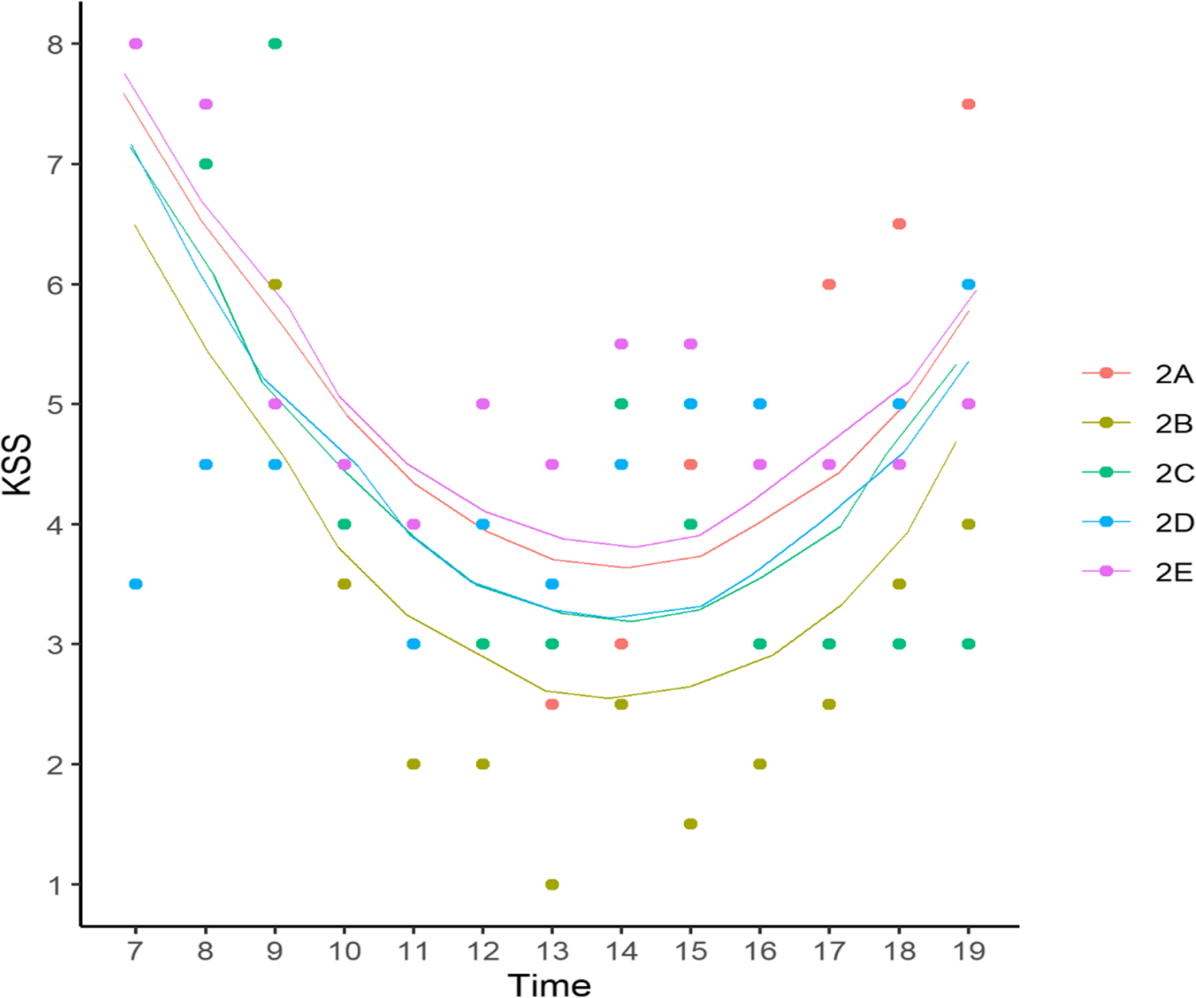
LMEM with Time of Day Predicting KSS on Day Two for each of the five participants in Study 1

On days one and two, both the first and second order terms predicted sleepiness (see Fig. 2 and Table 2). Participant *SD* on day one (0.43) and day two (0.57) were smaller than the corresponding residual variance *SD* in each LMEM (1.54 and 1.26). A significant curvilinear relation was shown, in which sleepiness fell from a morning high to a low around 2 p.m. and then increased in the evening.

**Table 2:** Statistics for generalised LMEMs for Study 1

Measure	Day	Model	Parameter	*b*	*β*	*SE b*	*t or z*	*p*	95% CI	AIC	BIC
KSS	One	First order	Time of day	-1.59	-3.42	.41	-3.92	< 0.001*	-2.38, -.79		
		Second order	Time of day	.06	3.27	.01	3.75	< 0.001*	.03, .08		
KSS	Two	First order	Time of day	-2.30	-4.85	.33	-6.97	< 0.001*	-2.95, -1.65		
		Second order	Time of day	.08	4.57	.01	6.56	< 0.001*	.06, .11		
Commission^	One	Linear	KSS	1.02	1.04	.03	.84	.40	.97, 1.07	332	338
		Quadratic	KSS	.96	.92	.15	-.30	.77	.70, 1.29	33	342
			KSS^2^	1.01	1.12	.01	.44	.66	.98, 1.04		
Commission^	Two	Linear	KSS	1.08	1.15	.03	2.79	.01*	1.02, 1.14	344	351
		Quadratic	KSS	.97	.95	.18	-.15	.88	.68, 1.39	346	355
			KSS^2^	1.01	1.20	.02	.58	.56	0.98, 1.04		
Mu	One	Linear	KSS	9.04	0.24	3.80	2.38	.02*	1.34, 16.43	689	698
		Quadratic	KSS	30.89	.84	20.66	1.50	.14	-9.68, 71.16	687	697
			KSS^2^	-2.20	-.60	2.05	-1.08	.29	-6.21, 1.8		
Mu	Two	Linear	KSS	-2.42	-.07	2.33	-1.04	.30	-7.06, 2.15	645	654
		Quadratic	KSS	8.79	.27	13.34	.66	.51	-17.84, 34.55	644	655
			KSS^2^	-1.12	-.34	1.31	-.85	.40	-3.65, 1.50		
Sigma	One	Linear	KSS	3.41	.25	1.42	2.41	.02*	.62, 6.20	566	575
		Quadratic	KSS	6.54	.48	7.76	.84	.40	-8.53, 21.85	566	577
			KSS^2^	-.31	-.23	0.77	-.41	.68	-1.83, 1.18		
Sigma	Two	Linear	KSS	3.01	.23	.72	4.16	< .001*	1.56, 4.43	500	509
		Quadratic	KSS	-1.82	-.14	4.14	-.44	.66	-9.96, 6.24	501	512
			KSS^2^	.48	.37	.41	1.18	.24	-.31, 1.28		
Tau	One	Linear	KSS	-2.16	-.13	1.80	-1.20	.24	-5.66, 1.50	596	605
		Quadratic	KSS	-11.37	-.67	9.81	-1.16	.25	-30.39, 8.07	596	606
			KSS^2^	.93	.55	.97	.95	.34	-.98, 2.82		
Tau	Two	Linear	KSS	.40	.03	.99	.41	.68	-1.53, 2.36	536	545
		Quadratic	KSS	-9.17	-.74	5.53	-1.66	.10	-19.83, 1.89	534	545
			KSS^2^	.95	.77	.541	1.76	.08	-.13, 2.00		

^Note. For the commission error, reported b coefficients were exponentiated values due to the use of GLMM. β indicates a standardised coefficient

### Hypothesis two: KSS and SART performance will have an inverted-U relation

**Fig. 3:**
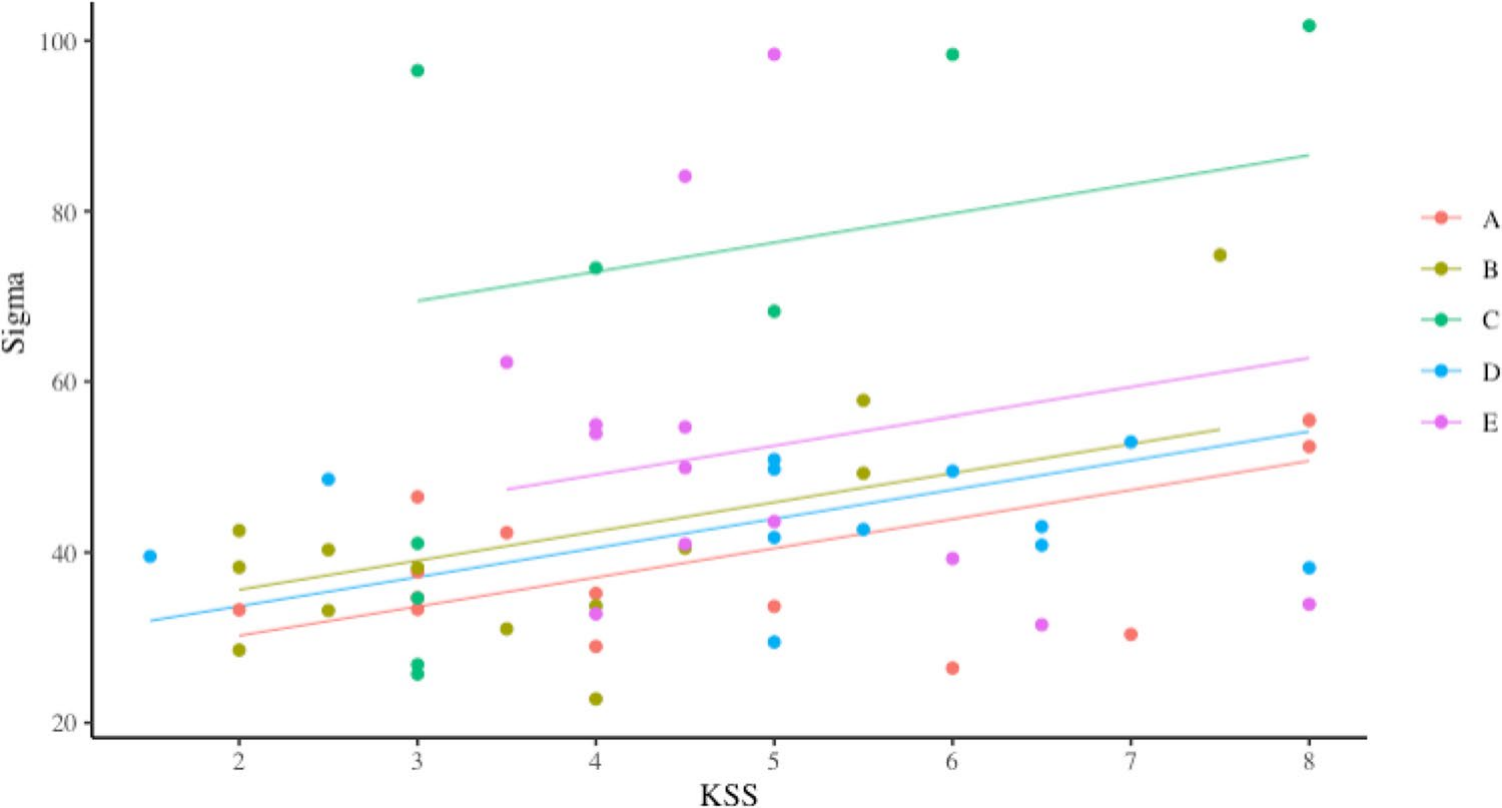
LMEM of KSS Predicting Sigma on Day One by Participant in Study 1

The results of the generalised LMEMs modelling the relation between KSS and each of the SART variables are presented in Table 2. For commission errors, only one model, the linear model from day two was significant. As KSS increased so did participant commission errors. The addition of the second-order polynomial term added no additional fitting benefit. For mu, only one model, the linear model from day one, was significant; response time slowed as participant sleepiness increased, however the change was not large. In the linear LMEM on day one, participant *SD* 44.79 was smaller than residual *SD* 50.39. Upon the addition of a second fixed effect, KSS^2^, the model was no longer significant. AIC and BIC statistics for the linear and quadratic variations each day were very similar. For sigma, the linear models for both day one and two were significant (see Fig. 3). Sigma increased as sleepiness increased. The quadratic models however were not significant on both days. In linear and quadratic LMEMs of day one, participant *SD* (15.22, 15.12) was smaller than the residual *SD* (18.78, 18.92). Goodness of fit statistics were too similar to be greatly informative. Little consistency was shown within participant sigma scores or in the way that sigma varied with sleepiness, across the two days. For tau, there were no significant associations with sleepiness.

## Study 1 discussion

The results of the small-*N* study supported the first hypothesis that time-of-day and arousal would have a curvilinear relation. Participants showed the predicted u-shape in sleepiness scores, in which sleepiness was high at 7 a.m., decreased until approximately 2 p.m. when sleepiness was lowest and then increased again towards the evening—clearly showing arousal consistently varying with time-of-day across the two days of the study. These findings support previous research demonstrating that arousal levels varied across time-of-day within a naturalistic study design, following a significant curvilinear pattern (Åkerstedt et al., 2013; Anderson & Revelle, 1994; Manly et al., 2002). While our observations align with Manly et al.’s (2002) finding that sleepiness was low at 1 p.m., our participants experienced a significant increase in sleepiness towards the evening, contrary to Manly et al.’s (2002) reports of equally high arousal at 1 p.m. and 7 p.m. Hypothesis two, stating that an inverted-u would be observed between each SART outcome measure and the sleepiness measure, was not supported. Some support was found for a linear relation predicting commission errors, mu, and sigma from the KSS scores, but the commission error and mu results were not significant. A linear relation between sigma and sleepiness was noted on both days. No significant relation was observed between tau and KSS scores. While arousal followed an inverted-u across time-of-day on both testing days, performance on the SART did not mirror this curvilinear variation. This study found no evidence supporting sustained attention performance being detrimentally impacted at the extremes of diurnal arousal variation.

To the best of our knowledge, no previous research has investigated sigma’s linear relation with sleepiness in a neurotypical sample. These results indicated that across the two days of testing, as sleepiness increased, so too did sigma—indicative of elevated rates of response time variations in participant responding. It is possible that such subtle variations in responding patterns are more vulnerable to arousal fluctuation than the other ex-Gaussian measures of tau and mu in healthy adults. Sigma may be a behavioural indicator of sleepiness.

Some significant linear relations were found between commission errors, mu and KSS, however their expression was variable across testing days. The finding of a significant linear relation with KSS and commission errors on day two supports results by Stawarczyk and D'Argembeau (2016) who reported a linear increase in commission errors as sleepiness rose. The lack of association on day one between commission errors and sleepiness aligns with findings by van Schie et al. (2014). As previous research investigating the relation between ex-Gaussian measures and arousal variation among a neurotypical population is scarce, mu and sigma are compared with previous measures of mean and standard deviation of response time; tau is not easily comparable. Findings of a significant linear relation with mu increasing (slowing) as KSS increased on day one contradict the findings of no relation between mean RT and sleepiness (Manly et al., 2002) or faster mean RT with increased sleepiness (Stawarczyk & D'Argembeau, 2016). The absence of a significant linear relation on day two between mu and KSS however was concordant with Manly et al. (2002) but not Stawarczyk and D'Argembeau (2016). The lack of a consistent significant relation across testing days between arousal, commission errors, and mu supports an interpretation that the true relation between these measures is minimal. To test whether the results of the small-*N* study were generalisable to the population a large-*N* sample study was conducted.

## Study 2: large-*N*

Contradictory findings in previous research of arousal’s varying effect on sustained attention performance outcomes warrants further investigation. With knowledge of the inverted-u distribution of optimal task performance across arousal levels (Aston-Jones & Cohen, 2005), a curvilinear relation might be expected to exist between subjective measures of sleepiness and SART performance measures. Study 1 had the benefit of testing an association between arousal levels and performance while controlling individual differences, but due to the sample size it lacked result generalisability to a general population. To address this disadvantage of Study 1, we further tested the same hypotheses in Study 2, which aimed to examine the relations between arousal and sustained attention using a large, more generalisable sample. The hypothesis was that an inverted-u relation would exist between KSS scores of subjective sleepiness and participant performance measures from the SART.

## Method

### Participants

Participants were sampled from friends and family and the School of Psychological Sciences Research Experience Program involving first year Psychology students at the University of Melbourne. Following ethics approval, informed consent was obtained from all participants prior to study commencement, and participants were fully debriefed. In total 200 participants were recruited. Of these participants, 12 participants were excluded due to making more than 30 omission errors in the SART and five failed to complete the study. A further 22 participants were excluded due to having existing mental health conditions, neurological conditions, severe head injury or for use of antidepressant, stimulant, or antipsychotic medication. The final sample was 161 participants (111 women, 49 men, two gender unspecified) whose ages ranged from 17 to 65 (*M* = 21.1 years, *SD* = 8.2). This study was conducted with the approval of the University of Melbourne Human Research Ethics Committee.

### Measures

The measures of Study 2 were identical to Study 1, aside from the version of the SART administered. In Study 2, the SART followed the design of Robertson et al. (1997), with each digit being presented 25 times pseudo-randomly across 225 trials and participants withholding the spacebar response to the digit 3.

### Procedure

This study was conducted online using the survey website Qualtrics and experimental administration program Inquisit to run the SART, during the period of COVID19 and lockdown orders. Email distribution was used to allocate participant numbers and a URL link to the Qualtrics survey. On Qualtrics, participants read and signed a plain language statement and consent form and confirmed that they would be in a quiet space free from distraction for the duration of the experiment, approximately 30 min. Participants then completed the demographic survey, ARCES, and CMQ before indicating their arousal as a KSS score, and they then downloaded the Inquisit player software used to run the SART on their personal computer. Following this approximately four-minute task, participants completed the second KSS and the CIQ. Once all tasks were complete participants read a debriefing statement.

### Data preparation

Questionnaire responses and SART data were prepared and analysed as described in Study 1. Regarding the KSS, minimal variation was seen in participant KSS scores before (*M* = 4.76, *SD* = 1.60) and after (*M* = 5.00, *SD* = 1.86) the SART, *t*(160) = 1.87, *p* = 0.06. Therefore, an averaged KSS score was calculated to provide an estimate of arousal for each testing session, for each participant.

Observing the distribution of CMQ scores, only three of 161 participants were outside the intermediate chronotype range. For this reason, analysis did not focus on CMQ as a factor. Omission error count was low and highly skewed therefore no further analysis was conducted with this measure.

### Statistical analysis

R version 3.5.2 (Team, 2018) and RStudio Version 1.3.1073 were used to analyse the data. Boxplots and quantile–quantile plots were used to assess normality and outliers within the data. Pearson’s and Spearman’s rho correlation coefficients were calculated between each SART outcome variable and the KSS. Generalised linear regressions with Poisson distribution were implemented for commission errors in accordance with analysing count data. Regressions for mu, sigma, and tau were conducted using linear regression models. To test the hypothesised inverted-u curve, the fit statistics of regressions with and without a second-order polynomial term for sleepiness were compared for each of the four dependent variables. Age was entered as a covariate due to the large variation across participants. Significance testing, adjusted *R*^2^, AIC and BIC were contrasted for the linear and non-linear (quadratic) regressions of commission errors, mu, sigma, and tau. Likelihood ratio (LR) testing further compared the proportion of variance explained by each regression.

## Results

### Descriptive statistics and correlations

Participant responses to questionnaire material and SART performance outcomes are presented in Table 3. Correlations were conducted to explore associations between the SART measures and the KSS, indicating small correlations—commission errors (*r* = 0.01), mu (*r* = 0.11), sigma (*r* = 0.15), and tau (*r* = 0.05)*.*

**Table 3:** Descriptive statistics of participant responses in Study 2

Measure^a^	*Mean*	*SD*	Range	
			Minimum	Maximum
ARCES^b^	2.89	.67	1.33	5
KSS	4.88	1.52	2	7.5
CIQ	2.24	.63	1.24	4.24
Mind Wandering^c^	3.80	1.47	1	7
Commission Errors	12.9	6.0	0	25
MRT (ms)	361	84	197	698
SDRT (ms)	106	57	35	396
Mu (ms)	281	90	101	693
Sigma (ms)	50	26	0	187
Tau (ms)	80	60	2	414

^a^ *N* = 161; ^b^ Attention-Related Cognitive Errors Scale mean score; ^c^ Item score on CIQ: Mind Wandering

### Hypothesis testing: comparative regressions

Table 4 presents the statistics of the regression models in which KSS scores and age predicted the different SART variables. Age was found to be a significant predictor of commission errors and mu, but not sigma or tau, in both linear and quadratic models. For all SART variable, neither the linear regression with a single predictor of KSS, or the quadratic regression with a second predictor of KSS^2^ were significant. Larger AIC and BIC values for each of the regression models with the second-order polynomial term indicated that its addition did not improve regression fit.

**Table 4:** Regression coefficients of KSS and age on the SART measures for Study 2

Measure	Model	*b*	*SE* of *b*	*β*	*p*	95% CI of *b*	Adjusted R^2^	AIC	BIC
Commission^	Linear							1167	1176
	Constant	19.95	0.112		< .001	16.03, 24.92			
	Age	0.99	0.015	.86	< .001*	0.97, 0.99			
	KSS	0.98	0.003	.98	.41	0.96, 1.02			
Commission^	Quadratic							1169	1181
	Constant	22.46	0.210		< 0.001	14.84, 33.80			
	Age	0.98	0.003	.86	< .001*	0.97, 0.99			
	KSS	0.94	0.084	.90	.42	0.79, 1.10			
	KSS^2^	1.01	0.009	1.09	.51	0.99, 1.02			
Mu	Linear				.02*		0.04	1904	1916
	Constant	200.23	32.14		< .001	136.75, 263.72			
	Age	2.39	0.86	.22	.01*	0.69, 4.10			
	KSS	6.15	4.64	.10	.19	-3.01, 15.31			
Mu	Quadratic				.04		0.03	1906	1921
	Constant	179.79	65.01		.01	51.37, 308.20			
	Age	2.38	0.87	.22	.01*	0.67, 4.09			
	KSS	15.73	26.86	.27	.56	-37.32, 68.79			
	KSS^2^	-1.00	2.80	-.17	.72	-6.43, 4.44			
Sigma	Linear				.19		.01	1508	1521
	Constant	44.16	9.40		< .001	25.58, 62.74			
	Age	-0.19	0.25	-.06	.46	-0.70, 0.31			
	KSS	2.06	1.36	.12	.13	-0.62, 4.74			
Sigma	Quadratic				.20		.01	1509	1525
	Constant	25.50	18.95		.18	-11.94, 62.94			
	Age	-0.20	0.25	-.06	.43	-0.70, 0.30			
	KSS	10.80	7.83	.64	.17	-4.67, 26.27			
	KSS^2^	-0.91	.80	-.52	.26	-2.50, 0.70			
Tau	Linear				.93		-.01	1782	1795
	Constant	85.02	22.02		< .001	41.54, 128.51			
	Age	0.02	0.59	.003	.97	-1.15, 1.19			
	KSS	-1.14	3.18	-.03	.72	-7.41, 5.14			
Tau	Quadratic				.99		-.02	1784	1800
	Constant	84.28	44.55		.06	-3.72, 172.28			
	Age	0.02	0.59	.003	.97	-1.15, 1.19			
	KSS	-0.79	18.40	-.02	.97	-37.15, 35.57			
	KSS^2^	-.04	1.89	-.01	.98	-3.77, 3.69			

^ Note. For the commission error, reported b coefficients were exponentiated values due to the use of GLMM. β indicates a standardised coefficient

## Study 2 discussion

The results of Study 2 did not support the hypothesised inverted-u relation between sustained attention performance and arousal. The lack of significant relation between KSS and commission errors was consistent with findings of van Schie et al. (2014), but contradict the findings of a negative impact of arousal variation on commission errors (Manly et al., 2002; Stawarczyk & D'Argembeau, 2016). The finding of no significant relation between mu and KSS scores aligned with conclusions of Manly et al. (2002) suggesting that average participant response speed is consistent over arousal variation.

When comparing the results of Studies One and Two, the only consistent result was the lack of association between tau and sleepiness. The significant linear relations reported in Study 1 between KSS and commission errors, mu, and sigma were not replicated in Study 2. In conclusion, there is very little evidence of a consistent association between arousal levels and sustained attention performance.

## General discussion

A significant inverted-u shape of arousal across time-of-day was reported in Study 1, providing additional support to previous research of curvilinear diurnal arousal variation (Valdez, 2019). Manipulating arousal by testing across different times of the day, instead of using sleep desynchrony programs or interventions, allowed greater insight into the natural fluctuation of arousal and concomitant variations in sustained attention performance. The results of these two studies demonstrated no support for the hypothesized inverted-u relation. Some linear relations between sleepiness and SART measures were reported in the small-*N* study, with repeated associations found for sigma, but no significant results for commission errors and mu, and no significant relation for tau. Much interest has been shown in what tau represents as a measure (Heathcote et al., 1991; Hohle, 1965; Leth-Steensen et al., 2000; Luce, 1986; Matzke & Wagenmakers, 2009). This study suggested that tau did not reflect underlying arousal levels within a participant. The only finding of a relationship between arousal and sustained attention performance in these studies was indicated by a linear association between sleepiness and sigma in Study 1. The hypothesised inverted-u relation, as recorded in primates by Aston-Jones and Cohen (2005) and suggested by Riley et al. (2017) in the human time-of-day and sustained attention work, was not shown. Nor was the linear relation between sleepiness and SART accuracy (Manly et al., 2002; Stawarczyk & D'Argembeau, 2016). Indeed, the current research showed little-to-no support for a linear relation between sleepiness and commission errors. In summary, arousal variation was linearly associated with sigma for the small-*N* study alone, and no other strong associations were noted between arousal and sustained attention over these two studies.

Having the capacity to sustain attention is a ubiquitous and important part of daily life that, when tested for about 5 min, appears unaffected by diurnal arousal variation. Participants reported KSS scores from 1 (*Extremely Alert*) to 8 (*Sleepy, Some Effort to Keep Alert*) across both studies. Nevertheless, accuracy and response speed on the SART did not significantly vary with reported KSS. Our study made no experimental manipulations to control or alter arousal levels except for the time of testing in Study 1, so arousal variation observed in typical lives were tested. This research suggested that although humans experience a range of sleepiness levels, this variation in sleepiness that individuals experience naturally has no relation to sustained attention performance.

A moderating factor may be the ability to self-motivate towards goals, enabling non-optimal arousal states to be countered by a cognitive desire to maintain accurate performance (Berridge & Arnsten, 2013). The work of Berridge & Arnsten, along with others (Åkerstedt et al., 2013; Anderson & Revelle, 1994) suggests that arousal undergoes stable daily fluctuations. To allow functioning to occur at any hour of the waking day our measures suggest that humans can override this sleepiness. This lack of association between diurnal arousal variation and SART performance suggests that concerns around testing time-of-day may be superfluous for a neurotypical adult sample. Over a range of arousal levels, participants seem able to compensate for sleepiness when asked to complete a sustained attention task. Arousal level might be critical and influence performance when it reaches more extreme levels such as sleep deprivation suggested by Manly et al. (2002), but this does not occur in typical daily lives. These results support a conclusion that arousal variation does not impact an individual’s sustained attention performance during the daylight hours.

There was a significant linear relation between sleepiness and sigma on both days of testing in Study 1, suggesting that this measure of response time variability is associated with arousal. This finding was not replicated in the large-*N* study however. We make two suggestions. First, the association between sigma and sleepiness may be specific to some individuals rather than a pattern seen in the general population. Second, it may be a nuanced signal that is observable when the experimental error term is curtailed under repeated measures conditions. Response time variability is argued to reflect general cognitive functioning (Carriere et al., 2010; Fortenbaugh et al., 2017; McAvinue et al., 2012). Many disorders are associated with increased response time variability (Tye et al., 2016; Vaurio et al., 2009). When sampled in a sample of cognitively healthy adults it is somewhat not surprising that sigma was not associated with sleepiness, but when the small sample underwent the SART 13 times a day, twice, the subtle signal between response time variability and sleepiness became apparent. Previous research using the SART and measures of sleepiness did not use such a detailed measure of response time variability as sigma. Further testing is needed to extricate information about this association.

Previous research on how arousal affects sustained attention in humans has predominantly used methodological designs in which arousal is experimentally manipulated through the implementation of a sleep desynchrony program or artificial stimulants (Harrison et al., 2007; Valdez et al., 2012). While such research is informative about performance at the extremes, it provides little insight into daily arousal variation and its influence on all tasks completed within waking hours. This current research is valuable as it provides further evidence demonstrating that arousal undergoes diurnal patterns—low in the morning, increasing towards peak levels in the early afternoon, then decreasing towards sleep. When aiming to evaluate performance, within experimental or every-day settings, using this variation to align activities with peak cognitive arousal may be useful. Within a neurotypical adult population, however, the arousal variations encountered across typical work hours do not impact an individual’s ability to sustain attention.

There are several limitations to this study. The first is that sustained attention performance was only measured over a relatively short period of time ~ 5.5 min. Variations in arousal might show stronger associations with sustained attention performance on more prolonged and difficult tasks, as shown by Stawarczyk and D'Argembeau (2016). Further studies should examine the effects of arousal on longer or more difficult versions of the SART or other prolonged vigilance tasks. Second, in both Study 1 and 2, participants were under lockdown orders related to the COVID-19 pandemic. Testing could only be conducted online, on each individual’s computer using online software. Participants would each have reacted differently to the strains of the pandemic, and we did not test stress, anxiety, or other related reactions to the situation that might have affected sustained attention performance. Third, some of the participants in Study 2 were sampled from family and friends and some were first year Psychology students and we note that these people may not be representative of the general population. Fourth, the intermediate range cut-off used in this study might not be age-appropriate for some of our participants. All of the participants in Study 1 and most in Study 2 scored in the intermediate range on the Composite Morningness Questionnaire (CMQ) and so this score was not used in further analyses. The intermediate range was calculated on an undergraduate student population (Smith et al., 1989). It is possible that the degree of diurnal preference changes with age and some of our participants were middle to older age. Although the average age of the participants in the CMQ was not published, we assume that the undergraduate student population was aged around 20 years of age. Future research might consider age-adjustments for the evening, morning, and intermediate chronotypes.

In conclusion, sustained attention is dependent on sufficient levels of cortical arousal (Calderon et al., 2016). Previous research investigating the consequence of arousal variation on sustained attention performance has been inconsistent. The results from this study demonstrated that arousal follows an inverted-u pattern across the course of a waking day. This diurnal variation, however, was not found to have an effect on sustained attention performance. Both the small-*N* and large-*N* studies found little support for a significant relation between varying arousal and SART performance. This finding refutes notions of performance being impacted by non-optimal arousal states, instead supporting a conclusion that the capacity to sustain attention withstands daily fluctuation in arousal.

## Data Availability

The datasets generated during and/or analysed during the current study will be available from the Open Science Framework.

## Code availability

The MATLAB script used is available at the Open Science Framework at https://osf.io/ntwy7/.

## Declarations

### Open practices statement

Data and materials for the experiments reported here will be available from the Open Science Framework. None of the experiments were preregistered.
